# Association of leisure-time sedentary behavior with fast food and carbonated soft drink consumption among 133,555 adolescents aged 12–15 years in 44 low- and middle-income countries

**DOI:** 10.1186/s12966-019-0796-3

**Published:** 2019-04-23

**Authors:** Garcia Ashdown-Franks, Davy Vancampfort, Joseph Firth, Lee Smith, Catherine M. Sabiston, Brendon Stubbs, Ai Koyanagi

**Affiliations:** 1 Department of Psychological Medicine, Institute of Psychiatry, Psychology and Neuroscience, King’s College London, De Crespigny Park, Box, London, SE5 8AF UK; 2 Department of Exercise Sciences, University of Toronto, 55 Harbord Street, Toronto, Ontario M5S 2W6 Canada; 3 Department of Rehabilitation Sciences, KU Leuven, Leuven, Belgium; 4 KU Leuven, University Psychiatric Center KU Leuven, Kortenberg, Belgium; 5 NICM Health Research Institute, Western Sydney University, Sydney, Australia; 6 Division of Psychology and Mental Health, Faculty of Biology, Medicine and Health, University of Manchester, Manchester, UK; 7 The Cambridge Centre for Sport and Exercise Sciences, Anglia Ruskin University, Cambridge, UK; 8 Physiotherapy Department, South London and Maudsley National Health Services Foundation Trust, London, UK; 9 Research and Development Unit, Parc Sanitari Sant Joan de Déu, CIBERSAM, Dr. Antoni Pujadas, Barcelona, Spain; 10 ICREA, Pg. Lluis Companys, 23 Barcelona, Spain

**Keywords:** Adolescents, Sedentary behavior, Fast food consumption, Soft drink consumption, Low- and middle-income countries (LMICs)

## Abstract

**Background:**

Rates of sedentary behavior (SB), fast food and carbonated soft drink consumption are increasing worldwide, with steeper increases being observed in low- and middle-income countries (LMICs) in recent years. Given that these behaviors have been linked to adverse health outcomes among adolescents, this presents a new but rapidly growing challenge to human health in these under-resourced nations. However, very little is known about the associations between SB and fast food or soft drink consumption among adolescents in LMICs.

**Methods:**

Thus, data from the Global school-based Student Health Survey (GSHS) were cross-sectionally analyzed in 133,555 adolescents aged 12–15 years from 44 LMICs [mean (SD) age 13.8 (1.0) years; 49% females]. The data were collected in the form of self-report questionnaires. Associations were assessed with multivariable logistic regression analysis and meta-analysis.

**Results:**

The overall prevalence of fast food consumption (at least once in previous 7 days) and carbonated soft drink consumption (at least once per day during past 30 days) were 49.3 and 43.8%, respectively. The overall pooled estimates based on a meta-analysis with random effects for the association of ≥3 h/day of SB with fast food consumption and soft drink consumption using country-wise estimates were OR = 1.35 (95% CI = 1.27–1.43, I^2^ = 62.1%).) and OR = 1.26 (95% CI = 1.19–1.34; I^2^ = 54.3%), respectively. Spending > 8 h/day of SB compared to < 1 h/day in females was associated with significantly higher odds for fast food (OR = 1.61, 95% CI = 1.38–1.88) and soft drink consumption (OR = 1.91, 95% CI = 1.60–2.28).

**Conclusions:**

Future interventions to address unhealthy behaviors in adolescents should take into account the interrelated nature of SB and unhealthy dietary habits, and seek to further understand the mechanisms linking these behaviors in the LMIC context.

## Background

Sedentary behavior (SB) (energy expenditure ≤1.5 metabolic equivalents of task (METs) while in a sitting or reclining posture during waking hours), is increasing rapidly in low and middle-income countries (LMICs) [24], owing to the introduction of both mechanized systems and digital technologies reducing the need for occupational and leisure-time physical activity [27]. There is increasing evidence that SB is associated with various negative health outcomes such as obesity, non-communicable diseases (NCDs) (e.g., diabetes, cardiovascular diseases, cancer) and premature mortality [4, 29]. These negative health outcomes of SB have been found throughout the life course including adolescence [4, 29]. For example, it has been found that SB such as television viewing during adolescence, is a key predictor of negative health outcomes such as being overweight, poor fitness and raised cholesterol during adulthood [18]. As such, the time frame of adolescence represents an essential window to prevent such health issues.

Among adolescents, recent studies have shown that the adverse health outcomes in SB may also be explained by poor dietary habits [14]. For example, previous studies from high-income countries have found that SB is associated with higher intake of snacks, sugar-sweetened beverages and fast foods [14], which are known risk factors for obesity and other negative health outcomes such as type-2 diabetes, hypertension and hypercholesterolemia and dental disease in adolescents [8, 26, 30].

While there are several studies that have found a positive association between higher levels of SB and poor dietary habits among adolescents from high-income countries [5, 14, 16, 34], there is a notable lack of studies from LMICs. This is an important research gap as the fast food industry (which arose from high-income countries) is now spreading across LMICs, introducing widespread consumption of ‘junk food’ and sugary carbonated drinks, both of which are strongly associated with increased rates of obesity and cardiometabolic mortality in these nations [35]. Indeed, there have been increases reported in both prevalence of fast food outlets, and frequency of fast food consumption in LMICs [22]. Relatedly, sales and consumption of soft drinks are increasing more rapidly in LMICs than in high-income countries [3]. Furthermore, there is a rapid increase in obesity and NCDs in LMICs mainly due to changes in lifestyles [15, 31], while almost 80% of deaths from non-communicable diseases occur in LMICs [37]. Thus, it is of vital importance to assess how unhealthy behaviors such as SB and consumption of fast food or sugar-sweetened soft drinks, which are increasing rapidly in LMICs, cluster in this context to counteract the NCD epidemic. Findings from high-income countries cannot be assumed to be automatically applicable to LMICs as there are differences in terms of accessibility to devices such as televisions and computers as well as to fast food restaurants. Thus, studies from a variety of settings are necessary for the development of context-specific public health interventions and policies. As such, the purpose of this study was to examine the relationship between SB, fast food consumption and carbonated soft drink consumption among adolescents in 41 LMICs.

## Methods

### The survey

Publicly available data from the Global School-Based Student Health Survey (GSHS) were analyzed. Details on this survey and the questionnaires can be found at http://www.who.int/chp/gshs and http://www.cdc.gov/gshs. Briefly, the GSHS was jointly developed by the World Health Organization (WHO) and the US Centers for Disease Control and Prevention (CDC), and other United Nations (UN) allies. The core aim of this survey was to assess and quantify risk and protective factors of major non-communicable diseases. The survey draws content from the CDC Youth Risk Behavior Survey (YRBS) for which test-retest reliability has been established [9]. The survey used a standardized two-stage probability sampling design for the selection process within each participating country. For the first stage, schools were selected with probability proportional to size sampling. The second stage involved the random selection of classrooms which included students aged 13–15 years within each selected school. All students in the selected classrooms were eligible to participate in the survey regardless of age. Data collection was performed during one regular class period. The questionnaire was translated into the local language in each country and consisted of multiple choice response options; students recorded their response on computer scannable sheets which were distributed by survey administrators. Students were instructed that completing the survey is voluntary and that questions can be left blank if they do not want to reply at the beginning of the survey. All GSHS surveys were approved, in each country, by both a national government administration (most often the Ministry of Health or Education) and an institutional review board or ethics committee. Student privacy was protected through anonymous and voluntary participation, and written informed consent was obtained as appropriate from the students, parents and/or school officials. Data were weighted for non-response and probability selection.

From all publicly available data, we selected all datasets that were nationally representative of students attending any type of school that included the variables pertaining to this analysis. High-income countries were excluded to focus on LMICs. If there were more than two datasets from the same country, we chose the most recent dataset. Thus, a total of 44 LMICs were included in the current study. For the included countries, the survey was conducted between 2009 and 2015, and consisted of 5 low-income, 26 lower middle-income, and 13 upper middle-income countries based on the World Bank classification at the time of the survey for the respective countries [36]. The list of countries included in the current study is provided in Table 1.

**Table 1:** Survey characteristics and prevalence of fast food consumption, carbonated soft drink consumption, and sedentary behavior

Country income level	Country	Year	Response rate (%)	N^a^	Fast food consumption (%)^b^	Soft drink consumption (%)^c^	Sedentary behavior (%)^d^
Low	Afghanistan	2014	79	1493	63.3	41.1	23.3
	Benin	2009	90	1170	51.8	32.1	18.4
	Cambodia	2013	85	1812	25.5	45.5	10.2
	Mozambique	2015	80	668	65.5	59.8	41.0
	Tanzania	2014	87	2615	35.6	47.6	20.1
	Total			14,786	48.3	47.8	21.2
Lower middle	Bangladesh	2014	91	2753	53.3	47.8	14.9
	Belize	2011	88	1600	66.2	63.9	36.3
	Bolivia	2012	88	2804	56.9	63.1	24.3
	East Timor	2015	79	1631	67.0	43.9	15.6
	Egypt	2011	85	2364	49.3	54.8	27.5
	El Salvador	2013	88	1615	57.4	65.9	35.2
	Ghana	2012	82	1110	69.9	55.2	18.4
	Guatemala	2015	82	3611	56.8	60.8	22.9
	Guyana	2010	76	1973	56.0	70.8	35.7
	Honduras	2012	79	1486	48.0	73.6	30.3
	Indonesia	2015	94	8806	54.7	29.2	24.5
	Kiribati	2011	85	1340	43.9	22.5	14.4
	Laos	2015	70	1644	44.8	58.2	19.2
	Maldives	2009	80	1981	34.9	32.8	42.4
	Mauritania	2010	70	1285	63.2	52.2	38.9
	Mongolia	2013	88	3707	55.2	33.1	39.6
	Morocco	2010	92	2405	44.2	46.3	25.7
	Pakistan	2009	76	4998	21.0	36.6	8.2
	Philippines	2015	79	6162	51.9	37.9	30.7
	Samoa	2011	79	2200	78.9	53.9	38.1
	Solomon Islands	2011	85	925	65.9	44.8	26.4
	Sudan	2012	77	1401	41.5	39.2	19.7
	Syria	2010	97	2929	42.8	31.1	25.3
	Tonga	2010	80	1946	70.0	57.3	29.2
	Vanuatu	2011	72	852	56.4	39.8	19.0
	Vietnam	2013	96	1743	29.7	34.6	34.9
	Total			86,957	48.3	40.0	24.5
Upper middle	Algeria	2011	98	3484	51.9	77.7	26.8
	Antigua & Barbuda	2009	67	1235	56.6	58.2	54.6
	Argentina	2012	71	21,528	31.5	66.0	49.9
	Costa Rica	2009	72	2265	54.4	52.6	44.2
	Iraq	2012	88	1533	55.7	53.9	25.6
	Lebanon	2011	87	1982	64.6	59.2	47.2
	Malaysia	2012	89	16,273	48.3	31.3	42.7
	Mauritius	2011	82	2074	54.2	39.5	39.2
	Namibia	2013	89	1936	53.9	51.4	37.2
	Peru	2010	85	2359	50.0	53.4	28.6
	Suriname	2009	89	1046	62.4	80.5	40.3
	Thailand	2015	89	4132	80.1	57.9	50.7
	Tuvalu	2013	90	679	44.5	54.0	15.2
	Total			84,792	56.7	56.7	39.3

^a^Restricted to those aged 12–15 years

^b^Fast food consumption referred to having eaten food from a fast food restaurant at least once in the past 7 days

^c^Soft drink consumption referred to drinking carbonated soft drinks at least once per day in the past 30 days

^d^Sedentary behavior referred to ≥3 h of sedentary time per day

### Sedentary behavior (SB)

SB was assessed with the question “How much time do you spend during a typical or usual day sitting and watching television, playing computer games, talking with friends, or doing other sitting activities?” with six response options: < 1, 1–2, 3–4, 5–6, 7–8, and > 8 h/day. This excluded time at school and when doing homework. This variable was used as a five-category variable (5–6 and 7–8 h/day were merged as the proportion of those who replied 7–8 h/day was small) or a dichotomized variable (≥3 h/day or not) [17]. This question was based on the National Health and Nutrition Examination Survey (NHANES) questionnaire from 1999 to 2000, and modified for use in children.

### Fast food consumption

Fast food consumption was assessed with the question “During the past 7 days, on how many days did you eat food from a fast food restaurant?” with country specific examples on fast food restaurants. The response options for this question were 0, 1, 2, 3, 4, 5, 6, or 7 days. This variable was dichotomized as at least once or not.

### Carbonated soft drink consumption

Consumption of carbonated soft drinks was assessed with the question “During the past 30 days, how many times per day did you usually drink carbonated soft drinks?” Country specific examples of carbonated soft drinks were provided (e.g., Carabao, Youki) and the student was instructed not to include diet soft drinks. Response options included ‘I did not drink carbonated soft drinks during the past 30 days’, ‘less than 1 time per day’, ‘1 time per day’, ‘2 times per day’, ‘3 times per day’, ‘4 times per day’, and ‘5 or more times per day’. This variable was dichotomized as ≥1 time per day or not.

### Control variables

Covariates included age, sex, food insecurity (proxy of socioeconomic status), and physical activity. As in a previous GSHS study, food insecurity was used as a proxy for socioeconomic status as there were no variables on socioeconomic status in the GSHS [2]. Specifically, this was assessed by the question “During the past 30 days, how often did you go hungry because there was not enough food in your home?” Response options were categorized as ‘never’, ‘rarely/sometimes’, and ‘most of the time/always’ [28]. To assess levels of physical activity, questions that represented the PACE+ Adolescent Physical Activity Measure [32] were asked. This measure has been tested for validity and reliability [32]. The questions asked about the number of days in which the respondent engaged in physical activity of at least 60 min during the past 7 days. This did not include physical activity during physical education or gym classes. Those who engaged in ≥5 days of at least 60 min of physical activity in a week were considered to have a sufficient amount of physical activity [17].

### Statistical analysis

Statistical analyses were performed with Stata 14.1 (Stata Corp LP, College station, Texas). The analysis was restricted to those aged 12–15 years. We used multivariable logistic regression analysis to estimate the association between SB (independent variable) and fast food or carbonated soft drink consumption (dependent variables) using the overall, sex-wise, and country-wise samples. The exposure variable was the five-category SB variable when the overall or sex-wise sample was used. However, for country-wise analyses, we used the dichotomized SB variable (i.e., ≥3 h/day or not) to obtain stable estimates, as the sample size in each country was small. In order to assess between-country heterogeneity in the association between SB and fast food or carbonated soft drink consumption, we calculated the Higgins’s *I*^2^ which represents the degree of heterogeneity that is not explained by sampling error with a value of < 40% often considered as negligible and 40–60% as moderate heterogeneity [19]. A pooled estimate was obtained by combining the estimates for each country into a random effect meta-analysis (overall and by country-income level).

All regression analyses were adjusted for age, sex, food insecurity (proxy of socioeconomic status), and physical activity with the exception of the sex-wise analysis which was not adjusted for sex. The analysis using the overall and sex-wise samples additionally adjusted for country as fixed effects by including dummy variables for each country in the model [28]. All variables were included in the regression analysis as categorical variables with the exception of age (continuous variable). Under 2.3% of the data were missing for the variables included in the study. Complete case analysis was done. Sampling weights and the clustered sampling design of the surveys were taken into account to obtain nationally representative estimates. In this study, we did not use multilevel models as such analyses can produce biased estimates when used with complex study designs [33]. Results from the logistic regression analyses are presented as odds ratios (ORs) with 95% confidence intervals (CIs). The level of statistical significance was set at *p* < 0.05.

## Results

### Sample characteristics and prevalence of fast food and carbonated soft drink consumption and leisure-time sedentary behavior

The final sample comprised 133,555 adolescents aged 12–15 years [mean (SD) age 13.8 (1.0) years; 49.0% females]. The characteristics of each country or survey are provided in Table 1. The average response rate across all countries was 83.5%. The overall prevalence of fast food consumption (at least once in previous 7 days) and carbonated soft drink consumption (at least once per day during past 30 days) were 49.3 and 43.8%, respectively. This prevalence varied widely between countries with the ranges being 21.0% (Pakistan) to 80.1% (Thailand) for fast food consumption and 22.5% (Kiribati) to 80.5% (Suriname) for carbonated soft drinks (Table 1). Overall, 27.0% of the adolescents engaged in ≥3 h/day of SB per day [range: 8.2% (Pakistan) to 54.6% (Antigua & Barbuda)]. The overall prevalence of SB were: < 1 h/day 38.7%; 1–2 h/day 34.3%; 3–4 h/day 15.8%; 5–8 h/day 7.7%; and > 8 h/day 3.6%.

### The association of fast food and carbonated soft drink consumption with leisure-time sedentary behavior

**Fig. 1:**
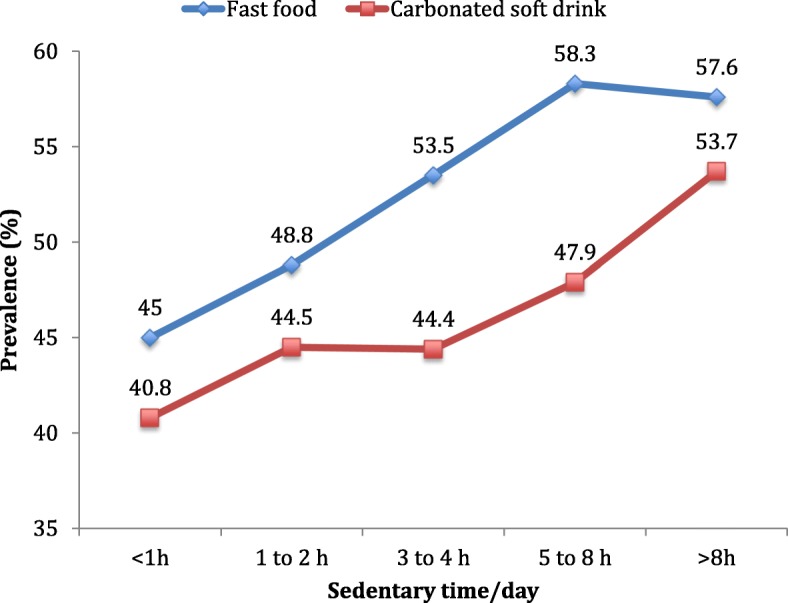
Prevalence of fast food and carbonated soft drink consumption by time spent sedentary per day. Fast food consumption referred to having eaten food from a fast food restaurant at least once in the past 7 days. Carbonated soft drink consumption referred to drinking carbonated soft drinks at least once per day in the past 30 days

The prevalence of fast food consumption and carbonated soft drink consumption increased with greater time spent sedentary per day (Fig. 1). This was also shown in the multivariable logistic regression analysis where compared to < 1 h/day of SB, the ORs (95% CIs) for fast food consumption and carbonated soft drink consumption were 1.45 (1.28–1.63) and 1.57 (1.37–1.81) for > 8 h/day of SB, respectively (Table 2). The associations were similar among boys and girls although the estimates for > 8 h/day of SB tended to be higher for girls.

**Table 2:** Association of time spent sedentary with fast food and carbonated soft drink consumption (outcomes) estimated by multivariable logistic regression

Fast food consumption^a^						
Time spent sedentary	Overall		Male		Female	
< 1 h/day	1.00		1.00		1.00	
1 to 2 h/day	1.16***	[1.08,1.25]	1.14*	[1.01,1.30]	1.17***	[1.07,1.28]
3 to 4 h/day	1.36***	[1.26,1.48]	1.34***	[1.19,1.51]	1.36***	[1.23,1.49]
5 to 8 h/day	1.54***	[1.36,1.75]	1.52***	[1.25,1.84]	1.51***	[1.31,1.74]
> 8 h/day	1.45***	[1.28,1.63]	1.27**	[1.06,1.52]	1.61***	[1.38,1.88]
Carbonated soft drink consumption^b^						
Time spent sedentary	Overall		Male		Female	
< 1 h/day	1.00		1.00		1.00	
1 to 2 h/day	1.17***	[1.09,1.25]	1.17**	[1.05,1.31]	1.15**	[1.04,1.26]
3 to 4 h/day	1.14***	[1.06,1.22]	1.15*	[1.03,1.28]	1.13*	[1.02,1.25]
5 to 8 h/day	1.24***	[1.10,1.40]	1.24*	[1.04,1.47]	1.21*	[1.05,1.39]
> 8 h/day	1.57***	[1.37,1.81]	1.29*	[1.05,1.60]	1.91***	[1.60,2.28]

Data are odds ratio [95% confidence interval]

Models are adjusted for age, socioeconomic status (food insecurity), physical activity, and country. Overall estimate is additionally adjusted for sex

^a^Fast food consumption referred to having eaten food from a fast food restaurant at least once in the past 7 days

^b^Carbonated soft drink consumption referred to drinking carbonated soft drinks at least once per day in the past 30 days

*p < 0.05, **p < 0.01, *** *p* < 0.001

**Fig. 2:**
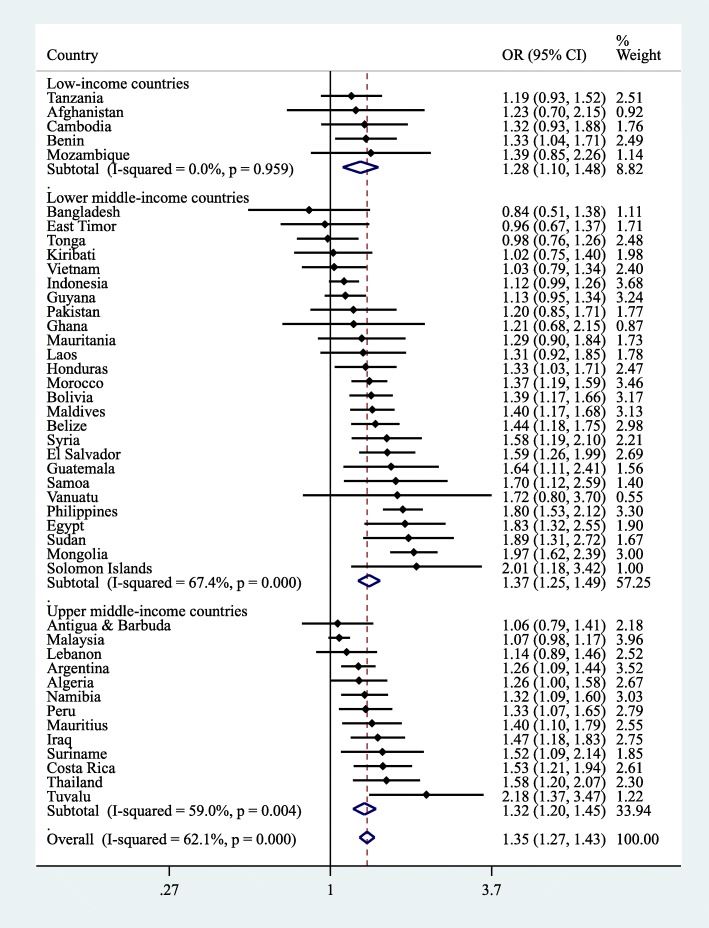
Country-wise association between ≥3 h/day of sedentary behavior and consumption of fast food estimated by multivariable logistic regression. Abbreviation: OR Odds ratio; CI Confidence interval. Models are adjusted for age, sex, socioeconomic status (food insecurity), and physical activity. Overall estimate is based on meta-analysis with random effects. Fast food consumption referred to having eaten food from a fast food restaurant at least once in the past 7 days

**Fig. 3:**
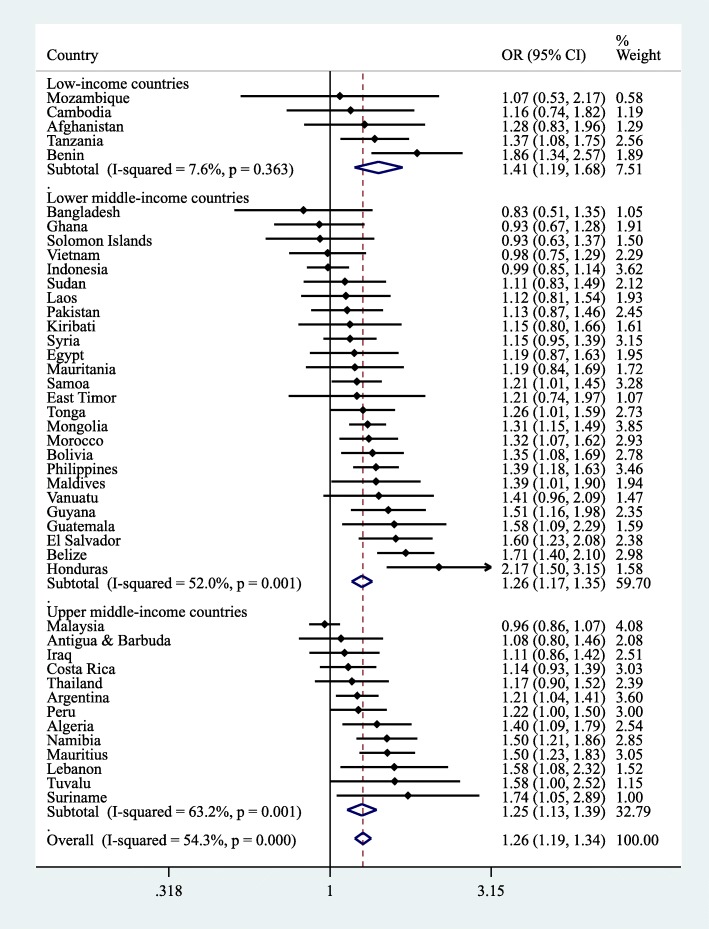
Country-wise association between ≥3 h/day of sedentary behavior and consumption of carbonated soft drink estimated by multivariable logistic regression. Abbreviation: OR Odds ratio; CI Confidence interval. Models are adjusted for age, sex, socioeconomic status (food insecurity), and physical activity. Overall estimate is based on meta-analysis with random effects. Carbonated soft drink consumption referred to drinking carbonated soft drinks at least once per day in the past 30 days

The country-wise associations between ≥3 h/day of SB and fast food consumption based on multivariable logistic regression are shown in Fig. 2. SB of ≥3 h/day was associated with higher odds for fast food consumption (i.e., OR > 1) in 41 of the 44 included countries with significant associations being observed in 25 countries. Particularly strong associations were observed in countries such as Tuvalu (OR = 2.18; 95% CI = 1.37–3.47), Solomon Islands (OR = 2.01; 95% CI = 1.18–3.42), and Mongolia (OR = 1.97; 95% CI = 1.62–2.39). The overall estimate based on a meta-analysis was OR = 1.35 (95% CI = 1.27–1.43) with a moderate level of heterogeneity being observed (*I*^*2*^ = 62.1%). The corresponding estimates for carbonated soft drink consumption are shown in Fig. 3. SB was associated with carbonated soft drink consumption in 38 countries with significant associations being observed in 22 countries. The strongest associations were observed in Honduras (OR = 2.17; 95% CI = 1.50–3.15), Benin (OR = 1.41; 95% CI = 1.19–1.68), and Suriname (OR = 1.74; OR = 1.05–2.89). The pooled estimate was OR = 1.26 (95% CI = 1.19–1.34; *I*^*2*^ = 54.3%).

## Discussion

### Main findings

To the best of our knowledge, this is the first multinational LMIC study to investigate the relationship between SB, fast food consumption and soft drink consumption in adolescents. We found that overall, 27% of adolescents spend ≥3 h/day of their leisure time being sedentary, 49% of adolescents had consumed fast food at least once within the past week, while 44% of adolescents had consumed a carbonated soft drink at least once per day in the past month. We also found that SB of ≥3 h/day was associated with higher odds for both fast food consumption and soft drink consumption in the majority of the 44 LMICs. Overall, a moderate level of between-country heterogeneity was observed but there were no major differences in the magnitude of the association by country income levels. Furthermore, consumption of fast foods and soft drinks appeared to increase with an increased time spent being sedentary.

### Interpretation

In general, the prevalence of fast food and soft drink consumption were comparable to high-income countries. For example, one multinational study from high-income countries found that frequent consumption of fast food (once or twice a week) among adolescents aged 13–14 years was 39% [7]. The overall prevalence of soft drink consumption was also similar to those previously reported from high-income countries [14]. This suggests that fast food and carbonated soft drink consumption can no longer be considered a problem of high-income countries alone. In addition, the fact that 27% of adolescents in our LMIC sample were sedentary for 3 h a day or more (excluding time spent at school or on homework) also highlight the importance of addressing health risk factors among adolescence in this context.

In our study, greater leisure-time SB was associated with greater fast food and carbonated soft drink consumption. This is in line with studies conducted in high-income countries. For example, among Australian adolescents, total screen time (i.e. not only television viewing) (OR = 1.80; 95% CI 1.33, 2.44, *p* < .05) and recreational computer use (OR = 1.50; 95% CI 1.13, 2.00, *p* < .05) were positively associated with weekly consumption of fast foods, while total screen time was also associated with greater consumption of sugar-sweetened beverages (OR = 1.11; CI 1.11–1.98, *p* < .05), as were television time and e-game time [14]. In a European sample (Greece, Norway, Hungary, Belgium, Spain, Switzerland), there was a positive association between mins/day of television viewing and mL/day of soft drink consumption. Interestingly, these results were found to be independent of individual and home environmental correlates of soft drink consumption, such as attitude towards soft drink consumption and parental modeling, respectively [16].

Fast food and carbonated soft drink consumption were both associated with time spent sedentary in the majority of the countries studied although a moderate level of between-country heterogeneity was observed. The reason for the moderate level of between-country heterogeneity is unknown but may be associated with factors such as difference in the content/context of SB or availability of fast food/carbonated soft drinks, or culture. For example, it has been established that youth from India, but also Lebanon are first exposed to fast foods through television commercials, and as such, differences in household ownership of televisions may play a role in this relationship [11, 23]. It has also been found in a European study that youth from lower-SES families are more likely to consume unhealthy drinks during television viewing [34]. Furthermore, it may be that changes in weather, temperature, access to poor foods and technological transitions might also be important factors [25].

The relationship between SB and soft drink or fast food consumption could be explained by factors such as mindless eating, advertising of fast foods and soft drinks during viewing times that are common for adolescents, or the use of popular youth programs as sponsors for fast foods and soft drinks [14]. Indeed, it may also be that computers and televisions act as a distractor, and that SB is accompanied by a cluster of other unhealthy behaviors such as the consumption of unhealthy food and drinks, and excessive SB may result in the creation of automatic cues for such dietary habits [6, 12]. It has been found that among adolescents, soft drink consumption and SB cluster together, and are related to each other in a habitual way, such that those who have a stronger habit of television viewing also have a stronger habit of soft drink consumption [12]. As such, the deleterious associations between SB and adverse physical health outcomes such as obesity may be at least partially accounted for by the co-occurrence with other unhealthy lifestyle behaviors such as fast food consumption [5]. Thus, also in LMICs, interventions may need to focus on addressing the habitual component of co-occurring carbonated soft drink/fast food consumption and SB.

### Policy implications and areas for future research

Taken together, our findings can be used to inform interventions targeted at decreasing SB, fast food and soft drink consumption among adolescents in LMICs. Importantly, although the associations between the three health behaviors were similar between sexes, the estimates for fast food and soft drink consumption with > 8 h/day of SB tended to be higher for girls. This suggests that girls may be in greater need than boys of intervention. Furthermore, these relationships appear to cluster together, so it may be that targeting one aspect, such as SB, will have positive outcomes in turn on fast food and soda drink consumption [13]. Future research and interventions should take into account the clustered nature of the relationship between SB, fast food consumption and soft drink consumption. Interventions using text messages as prompts to change health behaviors have been successful in the past (e.g. Cole-Lewis & Kershaw [10]). Evidence is emerging that also in LMICs mobile phones are an effective way to reach young people and to achieve knowledge and behavior change [21].

Given that the findings of the current study are similar to findings in high-income countries, many of which have guidelines or recommendations on limiting SB among adolescents (i.e., [1]), the creation of similar recommendations in LMICs may be warranted. Finally, as previous research has found that the majority of screen time occurs in the home and that most of the food adolescents consume is provided by their families, interventions are needed to encourage healthier eating habits while simultaneously discouraging sitting and television viewing, in the home context [14].

### Limitations

While this study provides novel findings especially in the LMIC context, it does have some limitations. First, although the overall response rate was high, there was some variation between countries with the response rates ranging from 67% (Antigua & Barbuda) to 98% (Algeria). Thus, it is possible that some level of bias was introduced in countries with low response rates. However, the use of sampling weights in the analysis is likely to have mitigated this potential bias. Second, the self-reported nature of the study may have resulted in adolescents inaccurately reporting their health behaviors, which may have biased the associations we found, and as such these findings must be taken in light of this. Third, the type and nature of SB were not measured. Past research has found that different types of SB may differentially affect food behaviors [14, 20]. For example, our study was on leisure-time SB and did not include SB during school time or while completing homework but it has been found that SB during homework completion has actually been linked to positive dietary behaviors such as increased fruit and vegetable consumption [20]. Relatedly, because students were instructed to exclude time spent at school or when doing homework when answering the question on time spent sedentary per day, this may have been difficult to calculate for some students. Furthermore, there may not have been many students who can spend > 8 h/day of SB out of school or when not doing homework and these students may have been a group with particular characteristics such as those attending schools with short schooling hours. In addition, only adolescents attending school were included in this study. Thus, our study results may not be generalizable to those who do not attend school. Finally, the cross-sectional nature of this study means that causation and directionality cannot be inferred.

## Conclusion

In conclusion, this is the first multinational LMIC study to investigate the relationship between SB, fast food consumption and soft drink consumption in adolescents. The results demonstrate that among adolescents in LMICs, rates of fast-food consumption and soft drink consumption increased with increasing time spent sedentary. There were some differences in the findings between countries and sexes, suggesting that context- or sex-specific strategies may be necessary. Future research is needed to confirm the causal aspects of this relationship and to specifically examine the exact context of SB and how this relates to unhealthy dietary habits among adolescents in LMICs for the establishment of effective strategies to reduce SB and poor dietary habits.
